# ConPlot: web-based application for the visualization of protein contact maps integrated with other data

**DOI:** 10.1093/bioinformatics/btab049

**Published:** 2021-01-28

**Authors:** Filomeno Sánchez Rodríguez, Shahram Mesdaghi, Adam J. Simpkin, J. Javier Burgos-Mármol, David L. Murphy, Ville Uski, Ronan M. Keegan, Daniel J. Rigden

**Affiliations:** Institute of Structural, Molecular and Integrative Biology, University of Liverpool, Liverpool L69 7ZB, UK; Life Science, Diamond Light Source, Harwell Science and Innovation Campus, Didcot, Oxfordshire OX11 0DE, UK; Institute of Structural, Molecular and Integrative Biology, University of Liverpool, Liverpool L69 7ZB, UK; Institute of Structural, Molecular and Integrative Biology, University of Liverpool, Liverpool L69 7ZB, UK; Institute of Structural, Molecular and Integrative Biology, University of Liverpool, Liverpool L69 7ZB, UK; Institute of Structural, Molecular and Integrative Biology, University of Liverpool, Liverpool L69 7ZB, UK; UKRI-STFC, Rutherford Appleton Laboratory, Research Complex at Harwell, Didcot OX11 0FA, UK; UKRI-STFC, Rutherford Appleton Laboratory, Research Complex at Harwell, Didcot OX11 0FA, UK; Institute of Structural, Molecular and Integrative Biology, University of Liverpool, Liverpool L69 7ZB, UK

## Abstract

**Summary:**

Covariance-based predictions of residue contacts and inter-residue distances are an increasingly popular data type in protein bioinformatics. Here we present ConPlot, a web-based application for convenient display and analysis of contact maps and distograms. Integration of predicted contact data with other predictions is often required to facilitate inference of structural features. ConPlot can therefore use the empty space near the contact map diagonal to display multiple coloured tracks representing other sequence-based predictions. Popular file formats are natively read and bespoke data can also be flexibly displayed. This novel visualization will enable easier interpretation of predicted contact maps.

**Availability and implementation:**

available online at www.conplot.org, along with documentation and examples. Alternatively, ConPlot can be installed and used locally using the docker image from the project’s Docker Hub repository. ConPlot is licensed under the BSD 3-Clause.

**Supplementary information:**

Supplementary data are available at *Bioinformatics* online.

## 1 Introduction

Recent developments in the field of evolutionary covariance have enabled increasingly accurate residue–residue contact predictions (e.g. Kandathil *et al.*, 2019) with wide utility in structural bioinformatics (de Oliveira and Deane, 2017) and structural biology (Simkovic et al., 2017). For example, they enable more accurate protein ab initio modelling (e.g. Zheng et al., 2019), identification of protein domain boundaries (Rigden, 2002; Sadowski, 2013), building search models for molecular replacement (Simkovic et al., 2016) and identification of similar local folds (Ovchinnikov et al., 2017). Classical representations of these predictions consist of two-dimensional binary matrices called contact maps (Godzik et al., 1993). These typically omit contacts between sequential near neighbours resulting in a blank space on and near the diagonal axis of the matrix.

A multitude of properties can be predicted by other sequence-based methods and researchers often need to consider diverse sources of information in order to form a complete and integrated picture. The diagonal of the contact map has been used in the past for secondary structure information (e.g. Taylor, 2016), but we are not aware of a tool to facilitate production of such images and, in any case, the typically empty space off-diagonal offers the possibility to display multiple tracks of data. Furthermore, although other interactive tools to work with contact maps have been developed (Kozma et al., 2012; Pietal et al., 2007; Vehlow et al., 2011) there currently seems to be no web-based application for convenient display and exploration of predicted contact maps and distograms.

Here we present ConPlot, the first tool of its kind, that presents sequence-based predictions in the form of multiple coloured data tracks near the diagonal axis of contact maps and distograms. This integration enables researchers to easily analyse a variety of data simultaneously and facilitates discovery of structural features.

## 2 Materials and methods

Written in *Python*, ConPlot is based on the *Dash* (Plotly Technologies Inc., 2020) web framework, which is an open-source Python library focused on the creation of interactive data visualization web sites. For data input, ConPlot has a parser module with functions to process a variety of commonly used sequence predictions and contact map formats plus the CASP RR RMODE 2 format of binned inter-residue distances.

Upon visiting the web application, users are assigned an unique universal identifier (UUID), which identifies their session until they leave the site and is used as a key to access data in a REDIS database (Redis Labs Inc., 2020). This database is used for the purpose of cache storage, and the assignment of these UUIDs ensures that data can only be accessed by the user who uploaded it. For long-term storage, optional account creation enables use of a persistent database, implemented using POSTGRESQL (Stonebraker et al., 2018), which can also be shared between collaborating registered users.

ConPlot is available at www.conplot.org, where it is deployed as a Docker container. Private use is possible using a Docker image from the project’s Docker Hub repository. Documentation and a set of tutorials are also accessible at the ConPlot website.

## 3 ConPlot features

ConPlot’s plots represent sequence-based predictions as coloured tracks displayed near the diagonal of the contact maps. Up to 9 tracks can be added to the diagonal, numbered from -4 to +4 according to their position relative to the diagonal track 0. These tracks are fully customizable regarding data, colour palettes and optional track mirroring across diagonal. The two halves of the contact map can be set to display different contact maps of matching sequence for comparison. The user can easily explore and interact with the data, zooming into different areas and hovering over data points to display detailed information.

For ease of use, ConPlot parses these popular file formats: PSIPRED secondary structure predictions (McGuffin et al., 2000), IUPRED sequence disorder predictions (Dosztányi, 2018), TOPCONS membrane topology predictions (Tsirigos et al., 2015) and CONSURF sequence conservation prediction (Ashkenazy et al., 2016). A custom data file format allows the user to display any other kind of information as a track. These custom files contain all the data required for ConPlot to create a coloured track in the form of a series of instructions with categorical information about the colour of the different sections of the additional track (see Supplementary Fig. S1 and the website help section).

Lastly, ConPlot can extract residue contact information from a PDB model and superpose predicted and model-derived contact maps: satisfaction of long-range contact predictions can then be used to infer model quality (de Oliveira et al., 2016; Miller and Eisenberg, 2008; Ovchinnikov *et al.*, 2017).

## 4 Use case

**Fig. 1.**
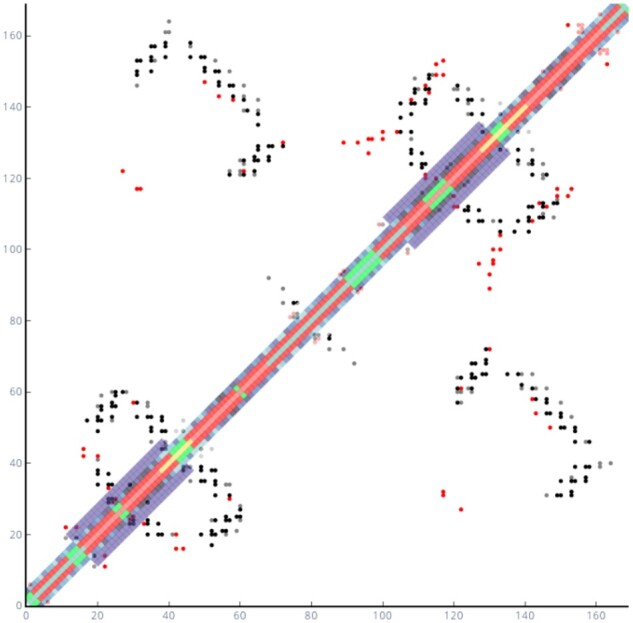
Superposition of DeepMetaPSICOV predicted contact map with contacts present in the structure modelled with DMPfold. Black points indicate matches between the two maps, red points indicate contacts present in the model but not predicted and grey points are contacts predicted but not present in the model. Central track 0 in the diagonal is used for the TOPCONS transmembrane prediction (blue—outside cell, yellow—inside cell, light red—predicted transmembrane helix). PSIPRED secondary structure prediction is visualized by the tracks +1 and -1 adjacent to the centre of the diagonal (red—helix, green—coil). Tracks +2 and -2 represent CONSURF sequence conservation prediction (blue gradient, darker blue—more conserved, lighter blue—less conserved). Outermost tracks +3, -3, +4 and -4 were added using a custom file in which the location of the suspected re-entrant loops is highlighted in purple: between residues 16–42 and residues 105–131. A companion figure illustrating the use of ‘Heatmap mode’ (for distograms or to illustrate contact prediction probabilities) is included as Supplementary Figure S4

To illustrate protein structural feature visualization from integrated contact and other predictions, we present analysis of a currently uncharacterized archaeal sequence (encoded by locus Mt2055 from Methanolobus tindarius; UniProt code W9DY28) from Pfam entry PF06695. Residue contact predictions were made using DeepMetaPSICOV (Kandathil *et al.*, 2019). Inspection in ConPlot, alongside transmembrane topology predictions from TOPCONS (Tsirigos *et al.*, 2015) and secondary structure predictions from PSIPRED (McGuffin *et al.*, 2000) revealed an unsuspected re-entrant loop structure between residues 16–42 (Fig. 1): a predicted transmembrane region (light red) with a break in the centre that separates two distinct predicted helices (dark red; from residues 16–25 and 28–42) in contact with each other (Yan and Luo, 2010) (Supplementary Fig. S2). CONSURF (Ashkenazy *et al.*, 2016) data (blue gradient) showed that this region is conserved and probably functionally important. Attempts to model the structure using the membrane topology prediction in conjunction with the RosettaMembrane protocol (Alford et al., 2015) were unsuccessful. However, models featuring a re-entrant loop which could be validated by their satisfaction of long-range contact predictions (Fig. 1) were eventually obtained using DMPfold (Greener et al., 2019) (Supplementary Fig. S3). The predicted contact map and models also highlight a second reentrant loop from residues 105–131, in accordance with weak evidence that the protein family resulted from a tandem duplication (Mesdaghi et al., 2020).

## 5 Conclusion

We present ConPlot, a new web-based application for the visualization of (predicted) protein contact maps alongside sequence annotations such as secondary structure predictions, transmembrane helical topology or sequence conservation. This juxtaposition facilitates structural analysis and prediction in the era of covariance-based contact predictions.

## Funding

This work was supported by the Biotechnology and Biological Sciences Research Council [BB/S007105/1].

*Conflict of Interest*: none declared.

## Supplementary Material

btab049_Supplementary_Data
