# Supplementary material for: ConPlot: web-based application for the visualization of protein contact maps integrated with other data
Source: Bioinformatics. 2021 Jan 28;37(17):2763–5. doi: 10.1093/bioinformatics/btab049 (PMC8428603; doi:10.1093/bioinformatics/btab049)
Supplement: btab049_Supplementary_Data [file btab049_supplementary_data.docx]

**Supplementary Figures**


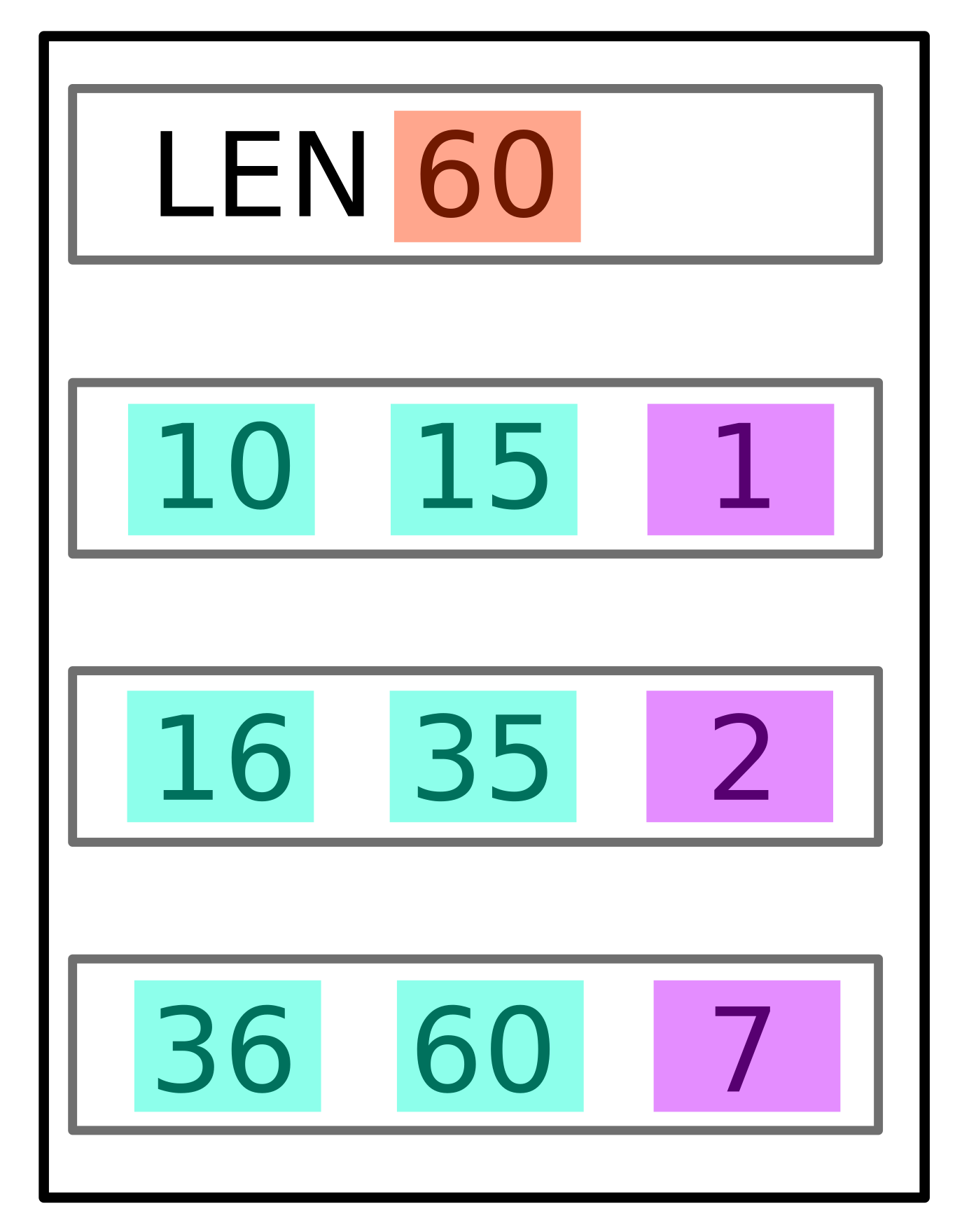


**Supplementary Figure 1.** Example of a ConPlot custom file. The first line of the file starts with the keyword “LEN”, followed by the length of the protein sequence that this file is intended to be used with (indicated with a red box). Subsequent lines inform about the colored tracks that the user wants to add, and consist of three fields. First and second field (turquoise boxes) indicate the start and the end residue numbers of the colored track (inclusive). The third field (purple boxes) corresponds with the color assigned to that particular track, which by default corresponds with a scale from 1 to 11 mapped to the full rainbow spectrum of colours. A secondary colour palette where this scale is mapped to a white-black spectrum is also available, and can be selected using ConPlot’s display settings interface.


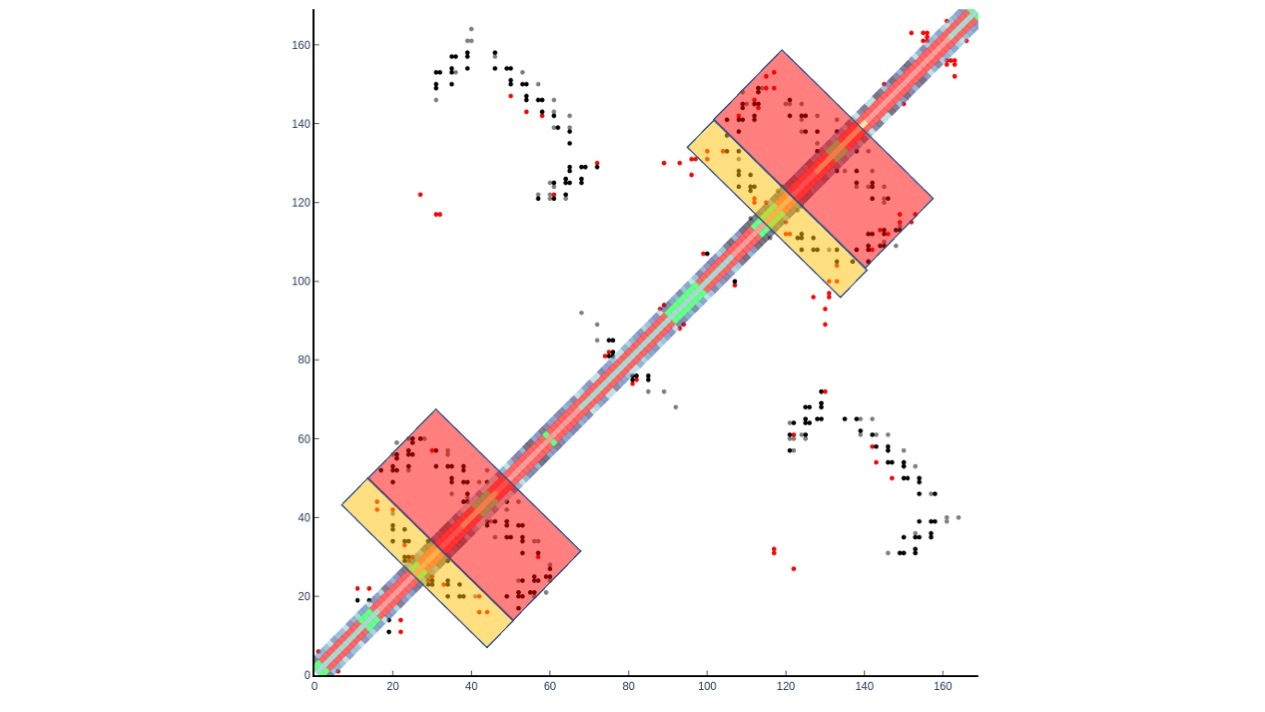


**Supplementary Figure 2.** Superposition of DeepMetaPSICOV predicted contact map with contacts present in the structure modelled with DMPfold. Colored boxes indicating the position of reentrant loop contacts (orange box) and contacts of TM helix packed with the reentrant loop (red box) have been added to the original plot produced by ConPlot. Black points indicate matches between the two maps, red points indicate contacts present in the model but not predicted and grey points are contacts predicted but not present in the model. Central track 0 in the diagonal is the ConPlot visual representation of the TOPCONS transmembrane prediction (blue - outside cell, yellow - inside cell, light red - predicted transmembrane helix). PSIPRED secondary structure prediction is visualised by the tracks +1 and -1 adjacent to the center of the diagonal (red - helix, green - coil). Outermost tracks +2 and -2 represent CONSURF sequence conservation prediction (blue gradient, darker blue - more conserved, lighter blue - less conserved).


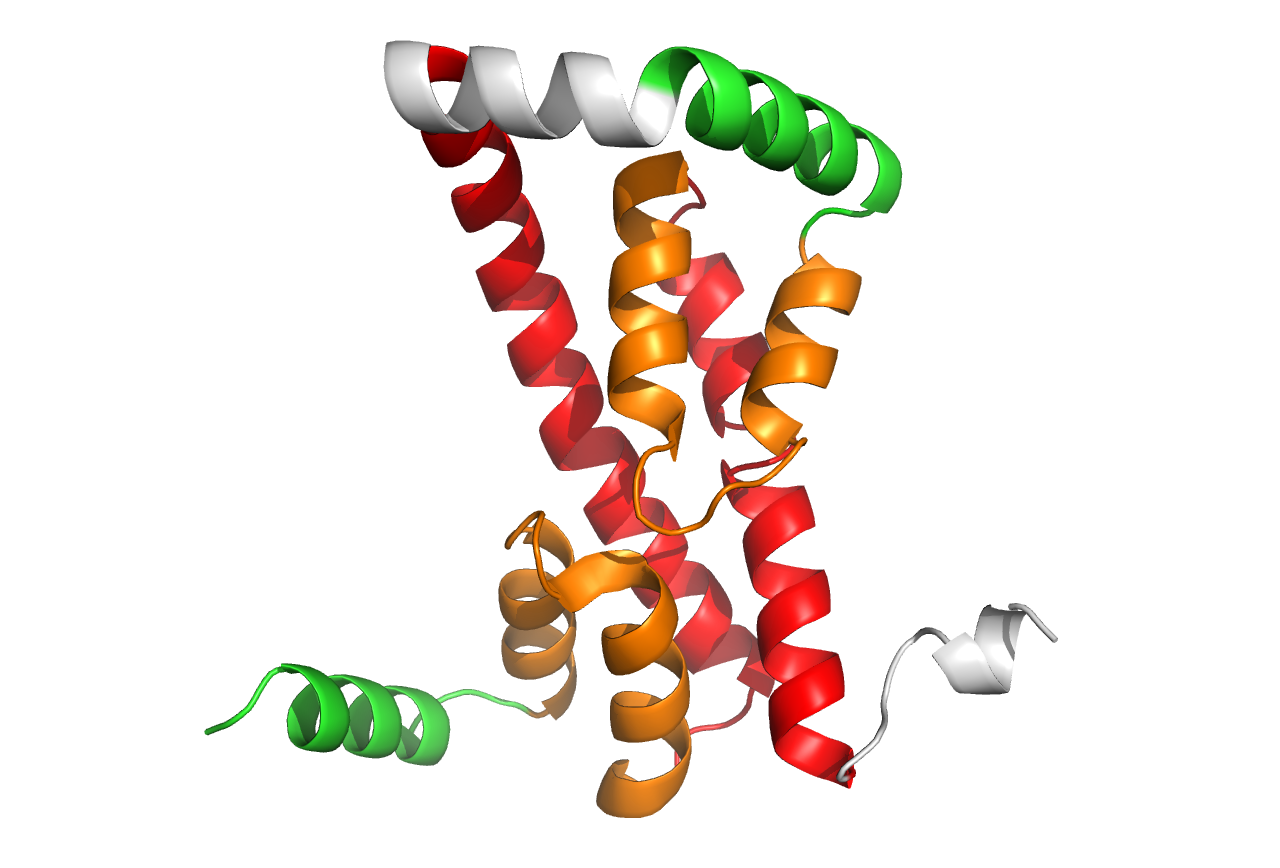


**Supplementary Figure 3.** DMPfold *ab initio* model of Mt2055. Orange regions are reentrant loops, red regions are TM helices, and green regions are amphipathic helices.

**
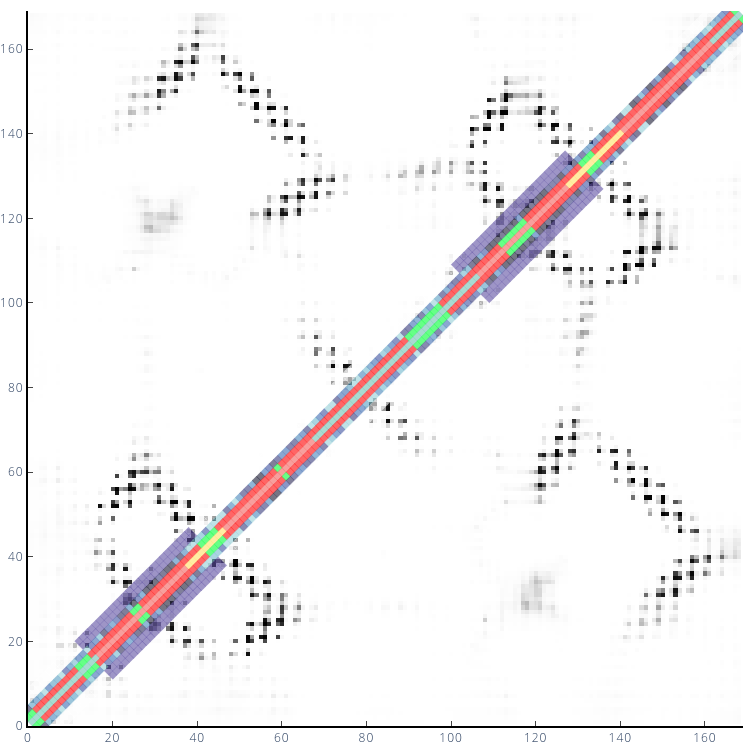
**

**Supplementary Figure 4. ‘**Heatmap mode’ representation of the DeepMetaPSICOV predicted contact map. Each contact is coloured according to its assigned confidence -the higher the confidence the darker the contact is shown. Central track 0 in the diagonal is used for the TOPCONS transmembrane prediction (blue - outside cell, yellow - inside cell, light red - predicted transmembrane helix). PSIPRED secondary structure prediction is visualised by the tracks +1 and -1 adjacent to the center of the diagonal (red - helix, green - coil). Tracks +2 and -2 represent CONSURF sequence conservation prediction (blue gradient, darker blue - more conserved, lighter blue - less conserved). Outermost tracks +3, -3, +4 and -4 were added using a custom file in which the location of the suspected re-entrant loops is highlighted in purple: between residues 16-42 and residues 105-131.
